# Implications of a Culturally Evolved Self for Notions of Free Will

**DOI:** 10.3389/fpsyg.2017.01889

**Published:** 2017-10-30

**Authors:** Lloyd Hawkeye Robertson

**Affiliations:** Integrated Studies, Athabasca University, La Ronge, SK, Canada

**Keywords:** self, free will, volition, culture, cultural evolution

## Abstract

Most schools in psychology have emphasized individual choice despite evidence of genetic and cultural determinism. It is suggested in this paper that the rejection of classical behaviorism by psychology and other humanities flowed from deeply held cultural assumptions about volition and free will. While compatibilists have suggested that notions of free will and determinism are not mutually exclusive, the psychological mechanisms by which such an accommodation could be explained have been inadequately explored. Drawing on research into classical cultures, this paper builds an argument that the notion of free will was adaptive flowing from culturally evolved changes to the self, and that this “evolved self,” containing assumptions of personal volition, continuity, and reason, became benchmarks of what it means to be human. The paper proposes a model of a culturally evolved self that is compatible with understandings of free will and determinism. Implications for therapeutic practice and future research are discussed.

## Introduction

The modern practice of psychology has been described as ethnocentric exporting Euro-American values such as individuality and self-volition to indigenous cultures worldwide (Adair, 2006; Christopher and Hickinbottom, 2008). The description of modern Euro-American cultures as “individualist” creates a dichotomy with more traditional cultures then viewed as collectivist. The self in modern western cultures is pictured as self-contained, independent, volitional, and materialistic while the self in collectivist cultures is described as contextual, interdependent, community orientated, and spiritual (Cushman, 1995; Robertson, 2014).

The concept of individual volition is necessarily grounded in notions of free will, that we are capable of choosing our behavior. While characterizations of “westerners” as individualist are not usually controversial, characterizations of non-westerners as groupist, collectivist, holist, or lacking in individual volition are increasingly seen as pejorative (Strauss, 2009). In response, Chirkov et al. (2003) suggested people could choose to identify with a collectivity and accept its norms, but their finding that a sample of university students drawn from four cultures (Turkey, Russia, Korea, and United States) scored higher on scales of well-being if they believed they voluntarily were part of a collective is also consistent with an interpretation that free will is a self-enhancing illusion (Taylor, 1989; Blackmore, 1999; Libet, 1999). It seems counterintuitive that a being constituted by cultural and genetic factors could transcend those very factors.

The purpose of this paper is to construct a paradigm by which a volitional individual could have evolved from determined beginnings. We begin by situating the self within the context of culture. This is followed by an examination of psychologists’ inherent epistemic volitional pre-suppositions. We conclude the body of this work with psycho-historical understanding of the evolution of the self-supporting a compatibilist account of free will.

## The Self As A Cultural Construct

The self that is core to such psychological concepts as self-esteem, self-actualization, and self-efficacy may be understood as a culturally mediated representational construct (Mead, 1934; Greenfield, 1995; Mischel and Morf, 2003). Although this cognitive self may be understood as a theory of who we are (Harre, 1989), it is not clear who or what constructs, holds or revises this theory leading to implicit dualism (Bhaskar and Norris, 1999; Seigel, 2005; Miresco and Kirmayer, 2006). Explicit dualism, wherein a stable conscious self is considered evidence of a non-corporeal entity inhabiting a material body, was the basis of the Cartesian notion that the physical world could be studied scientifically but the mind, which was not of this world, could not (Descartes, 1643/1990). Free will, from this dichotomization, is ultimately independent of material conditions because it is of supernatural origin.

Since the existence of a non-material body capable of holding the self has not been demonstrated, any argument for the existence of free will must, at this point of time, be based on a monist view of human development – the capacity for free will would have had to evolve in a being whose origins are material. For that reason, the self is defined here as a totality including physical, biological, psychological, social, and cultural characteristics (Quinn, 2006). This broad definition allows for: (1) the inclusion of unconscious factors that may influence behavior; (2) an evolutionary understanding of the development of the self built upon materialist beginnings; and, (3) the avoidance of a mind/body dualism implicit in a purely cognitivist view. If we take the view that human behavior was, at least in the first instance, determined by biological and environmental forces, then the self that emerged subsequent to this materialist foundation would continue to reflect that foundation.

The notion that the self and the volitional consciousness it is presumed to have are illusions received empirical support from Libet (1985) who demonstrated a neurological readiness to act in experimental subjects before they were consciously aware of making behavioral decisions to do so. Split brain experiments have demonstrated a mechanism whereby left hemispheric operations can give an interpretive after-the-fact but false causal rationalization for behavior (Sperry et al., 1969; Gazzaniga, 2000; Wolford et al., 2000). Drawing on such research, Blackmore (1999) declared the self to be a clever illusion by which a particular and arbitrary set of viral memes^1^ use consciousness, which is itself an “after the fact” illusion, to convince us that they are essential to our well-being. However, as Radder and Meynen (2013) noted, Libet’s (1985) original experimental design could not demonstrate that unconscious behavioral preparedness was a cause, a necessary condition, or even a correlation to subsequent behavior.

Dawkins (1976) declared that cultural memes have the capacity to attract or repel other memes. Identifying those attractive forces as connotative, affective, and behavioral associations held within minds with low replicative fidelity between individuals, Robertson (2010), mapped selves in a cross-cultural sample, and he used this mapping technique in psychotherapy (Robertson, 2011, 2016). He concluded that connotative, affective, and behavioral associations between memes lend stability to the representational structures so constructed. In the absence of dualism, a reasonable interpretation would be that such a mind had been appropriated by those memes so that the will of the individual is not his own but is reflective of the cultures from which those memes sprang. With the exception of one major school of psychotherapy, psychology has been resistant to this deterministic argument.

## Psychotherapy As A Secular Bastian of Free Will

According to Kuhn (1970a), psychology is a “proto-science,” not just for its failure to generate testable conclusions, but also for its failure to generate an overarching disciplinary paradigm under which such hypotheses could be generated. Classical behaviorists attempted to rectify this shortcoming by taking a determinist stance. Describing behaviorism as the philosophy of behavior science Skinner (1974, p. 14) explained, “The mentalistic problem can be avoided by going directly to prior physical causes while bypassing intermediate feelings or states of mind.” Chambless and Goldstein (1979, p. 232) elaborated, “Every behavior is considered to be completely determined by antecedent factors, leaving no room for cherished philosophical notions such as free will.” The self, in this view, is simply, “a functionally unified system of responses” (p. 238).

Behaviorists have used classical and operant conditioning to treat performance anxiety (Rodebaugh and Chambless, 2004), alcoholism (Trimpey, 1996), trauma (Devilly and Spence, 1999; Gerrity and Solomon, 2002), and conduct problems in children (Bloomquist and Schnell, 2002). Further, behaviorism alone has been shown to be as effective as composite cognitive-behavioral therapies (Jacobson et al., 1996; Warwar and Greenberg, 2000). While granting that behaviorism had demonstrated economy and efficacy, Hutcheon (1996, p. 461) concluded it had failed to become the dominant paradigm in psychology because it “attacks the very roots of our cultural assumptions.”

Psychotherapy has been based on notions of rational self-change leading to self-efficacy (Stefflre and Matheny, 1968; Bandura et al., 2001; David and Szentagotai, 2006), and current behaviorists have incorporated this cognitivist stance into their practices (Wilson, 2014). Collaborative approaches mandating client choice are embedded in the profession’s codes of ethics (Canadian Psychological Association, 2000; Sheppard and Schulz, 2007; Gauthier et al., 2012).

Using grounded theory to compare the content of 17 textbooks from separate schools of psychotherapy and 17 interviews with Inuit elders drawn from the Canadian Arctic, Korhonen (2002, p. 151) was surprised to find, “All the (Western/Modern) theorists believed in the interrelatedness of emotion, thought, and behavior, and in inseparability of the client from his social context.” She noted that the elements of good counseling involve learning about the client as a unique individual, developing counselor awareness of biases, gaining knowledge of the client in a social context, accepting the uniqueness of the individual, using appropriate verbal and non-verbal communication, using community resources and the client’s natural support systems, building a supportive client/counselor relationship, and working together to develop goals and interventions. She expressed greater surprise that Inuit (Canadian Eskimo) elders, in their counseling capacity, shared similar values concluding:

Three dominant themes about humanity emerge in the interviews: that human beings are essentially similar; that each person is nevertheless unique; and that humans are thinking beings whose ability to reason is their most important tool for long life. The elders all make clear their belief that humans seem to have an innate and individual core of personality, a unique self that influences action and thought. (p. 213).

In his project of mapping the self, Robertson (2010) found that adult participants from collectivist and individualist cultures who were not in therapy exhibited self-identities that included volition, constancy, feeling, distinctness, intimacy, and social interest noting that these qualities (or their absence) are typically addressed by therapists. While a determined being would not be expected to exhibit volition, constancy or distinctness, their presence in a “self” could have mental health benefits even in cultures designated as “collectivist.” A study involving 1,660 Chinese adolescents found that a majority (85%) had a belief in their own individual volition and they scored higher on scales of cognitive and affective well-being than those who did not share this self-belief (Li et al., 2016). Psychologists from a variety of schools have reported that approaches assuming individual volition and/or self-regulation to be effective in cross-cultural settings (Friere et al., 2005; Seligman et al., 2005; Deci and Ryan, 2008; Robertson et al., 2015).

While schools of psychotherapy may be non-directive with an emphasis on individual volition, it is still possible to picture the psychotherapist as part of a determining client environment. If the free will that appears to be reflected in individual volition is illusory, then the development of that illusion needs an evolutionary explanation. DiCarlo (2010) argued that the body’s neurotransmitters reward “an illusion of understanding, benefit and perceived control” (Memetic Equilibrium, para. 6) leading to the creation of primal religions during the Upper Paleolithic period. An alternative understanding would be that consciousness allowed our ancestors to better understand causality, and that this led to the exercise of at least a limited form of free will. This understanding also requires an evolutionary explanation.

## The Agentic Self As An Evolved Structure

While the conscious autobiographical self may be seen as a cultural construct, the broader definition of self used here accommodates an understanding that cognitive systems necessarily co-evolved with sensory organs to process raw experience (Trehub, 2007). For example, the acts of spatially situating experience, mirror recognition, and mimetic imitation each imply a rudimentary but evolving consciousness. The development of language allowed for the refinement and transmission of concepts. Gazzaniga (2000) described the left-hemisphere of the cerebral cortex as a mechanism evolved for language acquisition that acts as an “interpreter” in finding order. He suggested that such a thinking device, once it obtains productive answers to its questions, “cannot help but give birth to the concept of self. Surely one question the device would ask is, ‘Who is solving all these problems? Let’s call it *me*’-and away it goes!” (Gazzaniga, 2000, para. 16). Of course, this understanding pre-supposes the development of indexical pronouns along with an ability to manipulate the concepts generated in language.

The evolutionary route that takes an organism from dim self-awareness associated with the maintenance of homeostasis to a conscious self-description as a volitional problem solver is necessarily lengthy. Even the development of language cannot guarantee such a self-descriptor because the skill of using language for problem solving must be learned without, in the first instance, being taught. Once language based problem solving does occur it may not be immediately clear to the individual who is solving the problems. The “interpreter” needs to be interpreted into existence. Dennett (1991) concluded that memes provide “thinking tools” that allow for self-definition and human consciousness. Coming from a social constructionist perspective, Martin and Sugarman (2001, p. 105) suggested that with “appropriation and internalization, and the thinking and understanding they enable, the individual’s mode of being is transformed from one of pre-reflective activity to one in which reflective, intentional agency is possible.”

Self-definition requires units of culture to which the entity may self-reference. Such memes likely co-evolved with genes giving definition to our species (White, 1969/1990; Freidman and Sing, 2004), with the plausible result that the behaviors of our ancestors were determined by both cultural and genetic instructions. If humans were determined by memes in the manner suggested by Blackmore (1999, 2000), then the most successful of us would have been those who were able to reliably replicate such memes with fidelity and fecundity. In an experiment comparing autistic adults and non-autistics, Atran (2002), asked subjects to repeat common sayings such as “Let a thousand flowers bloom” or “To everything there is a season.” The autistic subjects repeated the memes with which they were presented more literally and exactly than non-autistics presented with the same memes. The non-autistic subjects would typically modify the information they were given in ways that showed interpretation or inference with the result that their answers displayed greater variance than the autistics. In a related experiment (Atran, 2002) Christians were asked to write the meanings of the 10 Commandments. Despite the subjects’ expectations, interpretations of the commandments showed considerable variation with little evidence of consensus. It is possible that our human ancestors were reliable replicators of culturally learned memes as postulated by Blackmore (1999), but “non-autistic modern humans have either a diminished ability to do so, or they have additional mental attributes interfering with this more primal replicative function” (Robertson, 2017, p. 357). As we shall see, this change could have occurred relatively recently.

Dahlsgaard et al. (2005) identified the core themes of justice, humanity, temperance, wisdom, and transcendence in the philosophical and religious traditions of China, South Asia, and the West^2^. Earlier, Jaspers (1951) had noted that the basis of these traditions flowed from the thoughts of Confucius, Lao Tzu, Buddha, Zarathustra, Moses, Homer, and Plato, all of whom lived during the same period of time (800 to 200 BCE). He named this epoch “the Axial Age” surmising, “This was when the man with whom we live today came into being (p. 135).” Mahoney (1991, pp. 29, 30) called this epoch “a time of turnings… of unprecedented reflective and spiritual activity… when humans first ‘formally’ discovered the universe within themselves and the powers of faith and reason.” What prompted this period of self-exploration?

Following his analysis of early Greek literature, Jaynes (1976) concluded that pre-Homeric Greeks were unable to exercise self-agency. Operating like Blackmore’s (1999) “meme machines,” they relied on imitating pre-programmed cultural responses to triggering events. Events that were not culturally anticipated led to stress induced hallucinatory symptoms which were often interpreted as divine messages. Following the examination of a series of neuro-imaging studies on hallucinatory patients, Rowe (2012, p. 503) observed, “The change from bicamerality to consciousness does not require genetic modification. The acquisition of a narrative self with and analog ‘I’ and a metaphor ‘me’ can be accomplished by culture and learning.”

After examining data from pre-1000 BCE Greek and Egyptian cultures, Johnson (2003) said the people in these early civilizations lacked minds. He defined mind to be an evolved cognitive program involving a capacity for objective beliefs, individual volition, and internally consistent thought. A temporally and spatially situated self-referencing such constructs could be learned and transmitted culturally.

An evolutionary perspective allows us to avoid the assumption that the self-emerged suddenly. For example, the widespread replication of the Paleolithic Acheulean hand ax (Johnson, 2003; Lycett, 2008) suggests that makers were able to hold the conceptual design of the ax in mind. Similarly, the common Paleolithic practice of burying useful or valuable objects along with the deceased (Duarte et al., 1999; Riel-Salvatore et al., 2001) suggests a projected identity that would need such objects. Referencing Paleolithic data, Montemayor and Haladjian (2017) argued that increasingly complex cognitive systems evolved with a capacity for guiding attention while more primal independent perceptual systems continued to operate. The self would be one such evolving cognitive system.

Much of the philosophical and religious thought flowing from the Axial Age dealt with concepts such as justice, humanity, and temperance reflecting the role of the individual in society. It is argued here that the emergence of individuals with a capacity for objective, volitional and internally consistent thought would have necessitated such a redefinition roles and this was a factor in the philosophical outpouring marking this period. For example, the Buddhist doctrine of “no-self” appears to be a reaction to a volitional self already present (Nishida, 1921/1990; Buddha, 1980; Rinpoche, 1993). Early Confucian thought dealt with the moral development of the self in relation to the collectivity (Wu, 2017). While the presence of people whose selves were programmed for individual volition might be potentially destabilizing to host collectivist societies, such individuals allow an increased range of responses to problems or opportunities and for time-delineated planning. Thus, societies that incorporated the services of such persons as leaders, sages, or oracles, would have a competitive advantage over their neighbors.

The concept of individual volition entails a being that is in some sense unique from others. If deliberating involves a future event, then the notion of volition requires a feeling of constancy that the individual may be present in the planned for future. The act of planning requires making choices from possible behaviors while predicting results. What constitutes evidence useful in making predictions may be idiosyncratic; however, once the concept of individual volition is operative, failure invites retrospective analysis. While a range of explanations for failure is generally possible, if a postulated need for increased objectivity leads to greater success in predicting results, then that behavior is reinforced. Free will is to be found in setting aside habitual behaviors in consideration of new possibilities. While the individual is never truly autonomous with respect to biological and cultural influences, and those “determinants” would create a statistically probability favoring certain behavioral responses, the consideration of alternatives in selecting those that best satisfy personal needs coupled with the ability to act on those choices would constitute acts of free will.^3^ As Racine (2017) explained:

Free will originates from the first person experience of the world and one’s self-constructed understanding of his or her agency in the course of action. Clearly, this understanding is socially constructed, shaped in interaction with others and influenced by cultural backgrounds that nourish interpretations of the phenomenon of agency. (p. 7).

This conceptualization of free will explains both the success of classical behaviorism in therapy and psychology’s overwhelming rejection of it. We remain, primarily, the determined beings that characterized most of our evolutionary history; but the idea of free will has become part of our self-definition as a species, and it has been the psychotherapists’ project to help clients achieve this ideal.

That ideal is attainable, at least in limited instances. For example, if we understand operative cultural, environmental, and genetic forces that direct our behavior and choose to act contrary to those forces, then we have exercised free will. Thus, true altruism without the expectation of social reward is possible.^4^ In principle, actions in accord with cultural or biological imperatives may also be examples of free will if we could have acted otherwise, but chose not to do so.

Robertson (2010) prepared self-maps of a woman from the interior of China illustrating both her active and deferent capacities. The woman acknowledged that she was capable of individual volition; however, she said that such activity was not valued in her culture. She experimented with making personal and educational decisions, but she returned to being deferent to the will of significant others because conducting the research necessary for making good decisions “takes too much time.” This decision likely led to behaviors that were indistinguishable from female countrymen who made no such conscious decision.

The example of the Chinese woman illustrates that it is possible to make an informed decision to conform to a limiting cultural injunction for reasons of perceived self-interest. Making informed choices on the basis of self-interest meets the definition of free will used here. While her decision was no doubt influenced by her upbringing, many people from the same cultural background have made a different decision (Li et al., 2016).

The example of the Chinese woman illustrates a limitation of this conceptualization of free will: We are called upon to make decisions every day, and do not have the time to adequately research and generate alternatives for each action. Yet, without such research, how is it possible to generate reasoned alternatives from which to decide? In western societies, we may choose to be deferent to advertising, peer pressure, political or religious ideology, racial prejudice, or other heuristics that have the potential of limiting our free will as much as deferring to our elders.

A requirement that each decision be adequately researched for the purpose of generating alternatives is too strict a rule for determining the exercise of free will. The self, as it has evolved, is a reflective project. We have the capacity to review past patterns of behavior and generate alternative scripts for implementation should similar circumstances arise, in effect, reprogramming ourselves. This sounds like the project of psychotherapy.

## Discussion

As we have seen, despite the presence of an alternative paradigm (behaviorism)^5^ with demonstrated efficacy, the practice of psychotherapy has been to promote client empowerment and volitional choice. The suggestion was made that our very definition of what it means to be human includes the ability to make willful choices thereby serving to preclude deterministic understandings. An evolutionary model was proposed whereby the development of an agentic self arose from materialist beginnings. Free will, defined as the ability to make meaningful choices within the frame of our cultural and environmental experience, appears as a spandrel^6^ to an adaptive cultural mutation incorporating individual volition or agency into the conscious self of individuals.

Conscious volition requires taking the self as an object (James, 1892/1999; Mead, 1934/2003; Leary and Tangney, 2003) with the resultant illusion of a homunculus operating independent of material and cultural constraints. Such a radical free will is not proposed here; however, to be self-aware is “to be able to conceive of one’s individual existence in an objective, as opposed to subjective, manner” (Lock, 1981/1990, p.220). Objectivity is also a goal of science. Sagan (1996, p. 27) observed, “...every time we exercise self-criticism, every time we test our idea against the outside world, we are doing science.” It is not necessary that decision-making be independent of physical and cultural determinants, but that the horizon of understanding and the ability to do otherwise be increased.

It would hold that every time a person changes a belief as a result of hypothesis testing, they are engaging in a reflexive project of self-definition. Since facts do not have the power to change belief as can be seen by the large numbers of people who reject evidence of global warming, the efficacy of vaccinations and the theory of evolution, a decision must to be made to accept new evidence before belief change is possible. Since this decision is made despite experiencing the pre-conditions that initially caused the false belief, such a decision would be an example of free will.

If we have developed the ability to exercise free will, then it should be possible to identify examples of behavior that do not comply with biological or cultural imperatives and examine the cognitive antecedents for such behavior. For example, the practice of celibacy stands in contradiction to a biological imperative to procreate. If the practice of celibacy is prescribed by a person’s station in a given culture, then that practice may be an example of a cultural imperative trumping a genetic one. If, on the other hand, the practice of celibacy was chosen without cultural sanction or reward, and if that choice was made as a deliberative as opposed to random act, then that would constitute an example of free will.

In principle, it would be possible to make a conscious and reasoned decision that conforms to a genetic or cultural imperative in the exercise of free will; however, differentiating such occurrences from those of determined behavior would be difficult. For example, an individual may believe he made a conscious free-willed decision to enter the priesthood; however, in examining such a claim we would need to consider the possibility that the decision was inevitable due to personal and cultural factors. If celibacy was a condition of entering the priesthood, then the decision to be celibate was not freely willed, although the decision to become a priest might be. Conversely, a priest may believe he made a decision to break a vow of celibacy after considerable reflection; however, that could be a retroactive attribution of causation. Were we to decide that a biological imperative led to this priest’s decision, there could still be room for the concept of free will if the priest had the capacity, on reflection, to veto that decision.^7^

Questions of free will could not have been asked by beings unable to visualize the concept. It is proposed here that this capacity flowed from changes to self-identity enhancing individual volition and that Axial Age philosophy was a response to the need to define and limit the role of these individuals in what had been more primal collectivist societies. This proposition could be falsified by referencing earlier civilizations that grappled with such questions. At some point, however, our ancestors attained the capacity to conceptualize free will. The “modern self” containing this conceptualization would have evolved from earlier selves with the implication that we retain many primal self-features. Thus, while a self-referencing autobiographical self is necessary for the notion of subjectivity and its conceptualized opposite, objectivity, modern humans do not necessarily, or even primarily, function at this level. None-the-less, the capacity to use reason and engage in conceptual thought, to situate ourselves temporally and engage in future planning, all of which is enhanced if not made possible by the modern self, are commonly considered to be distinguishing characteristics of being human and are part of a common paradigm informing the practice of psychotherapy.

Foucault (1982/1997, p. 246) said the individualist self is a modern European invention in contrast with the Medieval ideal where “The monk must have the permission of his director to do anything, even die. Everything he does without permission is stealing; there is not a single moment when the monk can be autonomous.” Cushman (1995) contrasted the 19th century Euro-American bourgeois self that was frugal and hardworking with a 20th century consumer self that was still individualist but “empty.” McCormick (1996) said psychology promoted a western autonomous self in contrast to cultures aboriginal to the Americas that emphasize interrelatedness. Christopher and Hickinbottom (2008) suggested Positive Psychology is ethnocentric for inviting individuals from collectivist cultures to describe what would constitute happiness for them as such a request pre-supposes individuality. Such generalizations contrasting a collectivist from an individualist self have been done in the absence of empirical studies describing the selves of persons from those cultures.^8^ This paper has argued that the selves of people from both individualist and collectivist cultures will share a similar structure that will commonly include such capacities as individual volition, distinctiveness, constancy, community, intimacy, interrelatedness, and reflection. From this perspective, differences between individualist and collectivist cultures are not to be found in the selves of their members but in the ideologies justifying power relationships governing self-expression. Thus, the monk in the Foucaultian example may have had a fully developed modern self but had learned to repress self-expression in the interest of religious orthodoxy. Further cross-cultural research describing the selves of persons is indicated.

This paper has argued that humans enact genetically and culturally determined sequences upon the presentation of triggering stimuli, but that at some point in human history, we also developed a capacity for individual volition and self-reflection. A memetic model was used to understand the algorithmic process such evolution could take. Absolute unencumbered free will was not considered possible in this monist view; however, the idea of free will coupled with an objective stance serves to increase our available options. By grounding an understanding of the self in a theoretical model compatible with both the practice of client empowerment and a naturalistic worldview, I have endeavored to enunciate a paradigm that has been developing in the practice of psychotherapy.

## Author Contributions

LHR conceived this work and was solely responsible for the acquisition, analysis, and interpretation of the data used. He was responsible for all drafts and revisions including final approval before publication. He has agreed to be accountable for all aspects of the work in ensuring that questions related to the accuracy or integrity of any part of the work are appropriately investigated and resolved.

## Conflict of Interest Statement

The author declares that the research was conducted in the absence of any commercial or financial relationships that could be construed as a potential conflict of interest.

## Footnotes

1 The term “meme” was coined by evolutionary biologist Richard Dawkins in 1976 to represent an elemental unit of culture that is self-replicating. This paper uses that definition without the implication that these units of culture have an active existence outside of the mind. In the view of Wilson (1999, p. 148), “the Dawkin’s ‘meme’ has gradually replaced such synonyms as ‘mnemotype,’ ‘idene,’ ‘sociogene,’ ‘concept,’ and ‘culturgen’ in describing this phenomena.”

2 Specifically, Dahlsgaard et al. (2005) examined early texts of Confucianism, Taoism, Buddhism, Hinduism, Athenian philosophy, Judaism, Christianity, and Islam.

3 Free will defined in this way may co-exist with determinism. If free will was defined as freedom from prior constraints and influences, then the two concepts would be incompatible.

4 The rejoinder from a determinist perspective that the altruist must have been acting on prior causal factors could be supported empirically if the determinants of a sufficiently large sample of altruists were identified concomitant with an examination of non-altruists showing that none of these determinant factors were present in their genetic or environmental backgrounds. Until that study is done, we must grant that true altruism is possible.

5 In Kuhnian terms (Kuhn, 1970b) classical behaviorism would be a pre-paradigmatic formulation since has not gained general acceptance in the discipline.

6 The term “spandrel” was appropriated from architecture by Gould and Lewontin (1979) to represent a byproduct of an adaptation that has no clear benefit for the organism’s fitness and survival, but is part of the adaptation’s “structure.”

7 Libet (1999) suggested that while his subjects had shown a readiness to act before making a conscious decision to do so, there was still time to veto that act before exhibiting the behavior. Similarly, for the determinist position to hold the decision to act would be made by environmental or genetic determinants before the individual was conscious of such a decision.

8 In his analysis of studies on family structure, human baby names, dog names, words in newspapers and social values, Ogihara (2017) suggested Japanese culture has become more individualistic over time. Resultant changes to the selves of the individuals affected by such changes could be measured see Robertson (2010).

## References

[B1] Adair J. G. (2006). “Creating indigenous psychologies: insights from empirical social studies of the science of psychology,” in *Indigenous and Cultural Psychology: Understanding People in Context* eds Kim U. Yang K.-S. Hwang K.-K. (New York, NY: Springer Science) 467–486. 10.1007/0-387-28662-4_21

[B2] Atran S. (2002). *In Gods we Trust: The Evolutionary Landscape of Religion.* New York, NY: Oxford University Press.

[B3] Bandura A. Caprara G. Barbaranelli C. Pastorelli C. Regalia C. (2001). Sociocognitive self-regulatory mechanisms governing transgressive behavior. *J. Pers. Soc. Psychol.* 80 125–135. 10.1O37//O022-3514.80.1.125 11195885

[B4] Bhaskar R. Norris C. (1999). *Roy Bhaskar Interviewed.* Available at: http://www.raggedclaws.com/criticalrealism/archive/rbhaskar_rbi.html [accessed January 20 2006].

[B5] Blackmore S. (1999). *The Meme Machine.* Oxford: Oxford University Press.

[B6] Blackmore S. (2000). The power of memes. *Sci. Am.* 283 64–69. 10.1038/scientificamerican1000-64 11011384

[B7] Bloomquist M. L. Schnell S. V. (2002). *Helping Children with Aggression and Conduct Problems: Best Practices for Intervention.* New York, NY: Guilford Press.

[B8] Buddha (1980). *The Teachings of Buddha* 137th Edn. Tokyo: Bukkyo Dendo Kyokai.

[B9] Canadian Psychological Association (2000). *Canadian Code of Ethics for Psychologists* 3rd Edn. Ottawa, ON: Canadian Psychological Association.

[B10] Chambless D. L. Goldstein A. J. (1979). “Behavioral psychotherapy,” in *Current Psychotherapies* 2nd Edn ed. Corsini R. J. (Itasca, IL: F. E. Peacock Publishers) 230–272.

[B11] Chirkov V. Ryan R. M. Kim Y. Kaplan U. (2003). Differentiating autonomy from individualism and independence: a self-determination theory perspective on internalization of cultural orientations and well-being. *J. Pers. Soc. Psychol.* 84 97–110. 10.1037/0022-3514.84.1.97 12518973

[B12] Christopher J. C. Hickinbottom S. (2008). Positive psychology, ethnocentrism, and the disguised ideology of individualism. *Theory Psychol.* 18 563–589. 10.1177/0959354308093396

[B13] Cushman P. (1995). *Constructing the Self, Constructing America: A Cultural History of Psychotherapy.* Cambridge, MA: Perseus.

[B14] Dahlsgaard K. Peterson C. Seligman M. E. (2005). Shared virtue: the convergence of valued human strengths across culture and history. *Rev. Gen. Psychol.* 9 203–213. 10.1037/1089-2680.9.3.203

[B15] David D. Szentagotai A. (2006). Cognitions in cognitive-behavioral psychotherapies; toward an integrative model. *Clin. Psychol. Rev.* 26 284–298. 10.1016/j.cpr.2005.09.003 16325974

[B16] Dawkins R. (1976). *The Selfish Gene.* Oxford: Oxford University Press.

[B17] Deci E. L. Ryan R. M. (2008). Facilitating optimal motivation and psychological well-being across life’s domains. *Can. Psychol.* 49 14–23. 10.1037/0708-5591.49.1.14

[B18] Dennett D. C. (1991). *Consciousness Explained.* Boston, MA: Little, Brown and Company.

[B19] Descartes R. (1643/1990). “Meditations on the first philosophy and the principles of philosophy,” in *From Sentience to Symbols: Readings on Consciousness* eds Pickering J. Skinner M. (Toronto, ON: University of Toronto Press), 10–20.

[B20] Devilly G. J. Spence S. H. (1999). The relative efficacy and treatment distress of EMDR and a cognitive-behavior trauma treatment protocol in the amelioration of posttraumatic stress disorder. *J. Anxiety Disord.* 13 131–157. 10.1016/S0887-6185(98)00044-9 10225505

[B21] DiCarlo C. (2010). *How Problem Solving and Neurotransmission in the Upper Paleolithic Led to the Emergence and Maintenance of Memetic Equilibrium in Contemporary World Religions.* Available at: https://politicsandculture.org/2010/04/27/how-problem-solving-and-neurotransmission-in-the-upper-paleolithic-led-to-the-emergence-and-maintenance-of-memetic-equilibrium-in-contemporary-world-religions/

[B22] Duarte C. L. MaurÃcio J. O. Pettitt P. B. Souto P. Trinkaus E. Van Der Plicht H. (1999). The early Upper Paleolithic human skeleton from the Abrigo do Lagar Velho (Portugal) and modern human emergence in Iberia. *Proc. Natl. Acad. Sci. U.S.A.* 96 7604–7609. 10.1073/pnas.96.13.7604 10377462 PMC22133

[B23] Foucault M. (1982/1997). “Technologies of the self,” in *Ethics: Subjectivity and Truth* Vol. 1 ed. Rabinow P. Hurley trans. R. (New York, NY: New Press) 223–252.

[B24] Freidman D. Sing N. (2004). Negative reciprocity: the co-evolution of memes and genes. *Evol. Hum. Behav.* 25 155–173. 10.1016/j.evolhumbehav.2004.03.002

[B25] Friere E. S. Koller S. H. Piason A. da Silva R. B. (2005). Person-centered therapy with impoverished, maltreated and neglected children and adolescents in Brazil. *J. Ment. Health Couns.* 27 225–237. 10.17744/mehc.27.3.6p6qm84wqkkxp2a5

[B26] Gauthier J. Pettifor J. Ferrero A. (2012). The Universal Declaration of Ethical Principles for psychologists: a culture-sensitive model for creating and reviewing a code of ethics. *Ethics Behav.* 20 179–196. 10.1080/10508421003798885

[B27] Gazzaniga M. S. (2000). Cerebral specialization and interhemispheric communication: does the corpus callosum enable the human condition? *Brain* 137 1293–1326. 10.1093/brain/123.7.1293 10869045

[B28] Gerrity E. T. Solomon S. D. (2002). “The treatment of PTSD and related stress disorders: current research and clinical knowledge,” in *Ethnocultural Aspects of Posttraumatic Stress Disorder: Issues, Research, and Clinical Applications* eds Marsella A. J. Friedman M. J. Gerrity E. T. Scurfield R. M. (Washington, DC: American Psychological Association) 87–104.

[B29] Gould S. J. Lewontin R. C. (1979). The spandrels of San Marco and the Panglossian paradigm: a critique of the adaptationist programme. *Proc. R. Soc. Lond. B Biol. Sci.* 205 581–598. 10.1098/rspb.1979.0086 42062

[B30] Greenfield S. (1995). *Journey to the Centers of the Mind.* New York, NY: W.H. Freeman & Co.

[B31] Harre R. (1989). “The self as a theoretical concept,” in *Relativism: Interpretation and Confrontation* ed. Krausz M. (Notre Dame, IN: University of Notre Dame Press) 389–411.

[B32] Hutcheon P. D. (1996). *Leaving the Cave: Evolutionary Naturalism in Social-scientific thought.* Waterloo, ON: Wilfred Laurier University Press.

[B33] Jacobson N. S. Dobson K. S. Truax P. A. Addis M. E. Koerner K. Gollan J. K. (1996). A component analysis of cognitive-behavioral treatment for depression. *J. Consult. Clin. Psychol.* 64 295–304. 10.1037/0022-006X.64.2.295 8871414

[B34] James W. (1892/1999). “The self,” in *The Self in Social Psychology: Key Readings in Social Psychology* ed. Baumeister R. F. (New York, NY: Psychology Press) 69–77.

[B35] Jaspers K. (1951). *Way to Wisdom: An Introduction to Philosophy.* New Haven, CT: Yale University Press.

[B36] Jaynes J. (1976). *The Origins of Consciousness in the Breakdown of the Bicameral Mind.* Boston, MA: Houghton Mifflin.

[B37] Johnson D. M. (2003). *How History made Mind: The Cultural Origins of Objective Thinking.* Chicago, IL: Open Court Books.

[B38] Korhonen M.-L. (2002). *Inuit Clients and the Effective Helper: An Investigation of Culturally Sensitive Counselling.* Durham: University of Durham.

[B39] Kuhn T. S. (1970a). “Reflections on my critics,” in *Criticism and the growth of Knowledge* eds Lakatos I. Musgrave A. (London: Cambridge University Press) 231–278. 10.1017/CBO9781139171434.011

[B40] Kuhn T. S. (1970b). *The Structure of Scientific Revolutions.* Chicago, IL: University of Chicago Press.

[B41] Leary M. Tangney J. P. (2003). “The self as an organizing construct in the behavioral and social sciences,” in *Handbook of Self and Identity* eds Leary M. Tangney J. P. (New York, NY: Gilford Press.) 3–14.

[B42] Li C. Wang S. Zhao Y. Kong F. Li J. (2016). The freedom to pursue happiness: belief in free will predicts life satisfaction and positive affect among Chinese adolescents. *Front. Psychol.* 7:2027. 10.3389/fpsyg.2016.02027 28101072 PMC5209362

[B43] Libet B. (1985). Unconscious cerebral initiative and the role of conscious will in voluntary action. *Behav. Brain Sci.* 8 529–566. 10.1017/S0140525X00044903

[B44] Libet B. (1999). Do we have free will? *J. Conscious. Stud.* 6 47–57.

[B45] Lock A. (1981/1990). “Universals in human conception,” in *From Sentience to Symbols: Readings on Consciousness* eds Pickering J. Skinner M. (Toronto, ON: University of Toronto Press) 218–223.

[B46] Lycett S. J. (2008). Acheulean variation and selection: does handaxe symmetry fit neutral expectations? *J. Archaeol. Sci.* 35 2640–2648. 10.1016/j.jas.2008.05.002

[B47] Mahoney M. J. (1991). *Human Change Processes: The Scientific Foundations of Psychotherapy.* New York, NY: Basic Books.

[B48] Martin J. Sugarman J. (2001). Is the self a kind of understanding? *J. Theory Soc. Behav.* 31 103–114. 10.1111/1468-5914.00148

[B49] McCormick R. (1996). Culturally appropriate means and ends of counselling as described by the First Nations people of British Columbia. *Int. J. Adv. Couns.* 18 163–172. 10.1007/BF01407960

[B50] Mead G. H. (1934). *Mind, Self and Society.* Chicago, IL: University of Chicago Press.

[B51] Mead G. H. (1934/2003). “The self,” in *Inner Lives and Social Worlds: Readings in Social Psychology* eds Holstein J. A. Gubrium J. F. (New York, NY: Oxford University Press) 125–130.

[B52] Miresco M. J. Kirmayer L. J. (2006). The persistence of mind-brain dualism in psychiatric reasoning about clinical scenarios. *Am. J. Psychiatry* 163 913–918. 10.1176/ajp.2006.163.5.913 16648335

[B53] Mischel W. Morf C. C. (2003). “The self as a psycho-social dynamic processing system: a meta-perspective on a century of the self in psychology,” in *Handbook of Self and Identity* eds Leary M. Tangney J. P. (New York, NY: Gilford Press) 15–43.

[B54] Montemayor C. Haladjian H. H. (2017). Perception and cognition are largely independent, but still affect each other in systematic ways: arguments from evolution and the consciousness-attention dissociation. *Front. Psychol.* 8:40. 10.3389/fpsyg.2017.00040 28174551 PMC5258763

[B55] Nishida K. (1921/1990). *An Inquiry into the Good* Abe trans. M. Ives C. London: Yale University Press.

[B56] Ogihara Y. (2017). Temporal changes in individualism and their ramification in Japan: rising individualism and conflicts with persisting collectivism. *Front. Psychol.* 8:695. 10.3389/fpsyg.2017.00695 28588512 PMC5440576

[B57] Quinn N. (2006). The self. *Anthropol. Theory* 6 362–384. 10.1177/1463499606066893

[B58] Racine E. (2017). A proposal for a scientifically-informed and instrumentalist account of free will and voluntary action. *Front. Psychol.* 8:754. 10.3389/fpsyg.2017.00754 28567025 PMC5434413

[B59] Radder H. Meynen G. (2013). Does the brain “initiate” freely willed processes? A philosophy of science critique of Libet-type experiments and their interpretation. *Theory Psychol.* 23 3–21. 10.1177/0959354312460926

[B60] Riel-Salvatore J. Clark G. Davidson I. Noble W. DErrico F. Vanhaeren M. (2001). Grave markers: middle and Early Upper Paleolithic burials and the use of chronotypology in contemporary Paleolithic research 1. *Curr. Anthropol.* 42 449–479. 10.1086/321801

[B61] Rinpoche S. (1993). *The Tibetan Book on Living and Dying.* New York, NY: Harper Collins.

[B62] Robertson L. H. (2010). Mapping the self with units of culture. *Psychology* 1 185–193. 10.4236/psych.2010.13025

[B63] Robertson L. H. (2011). Self-mapping in treating suicide ideation: a case study. *Death Stud.* 35 267–280. 10.1080/07481187.2010.496687 24501846

[B64] Robertson L. H. (2014). In search of the aboriginal self: four individual perspectives. *Sage Open* 4 1–13. 10.1177/2158244014534246

[B65] Robertson L. H. (2016). Self-mapping in counselling: using memetic maps to enhance client reflectivity and therapeutic efficacy. *Can. J. Couns. Psychother.* 50 332–347.

[B66] Robertson L. H. (2017). The infected self: revisiting the metaphor of the mind virus. *Theory Psychol.* 27 354–368. 10.1177/0959354317696601

[B67] Robertson L. H. Holleran K. Samuels M. (2015). Tailoring university counselling services to aboriginal and international students: lessons from native and international student centres at a Canadian university. *Can. J. High. Educ.* 45 122–135.

[B68] Rodebaugh T. L. Chambless D. L. (2004). Cognitive therapy for performance anxiety. *J. Clin. Psychol.* 60 809–821. 10.1002/jclp.20039 15241809

[B69] Rowe B. (2012). Retrospective: Julian Jaynes and the origin of consciousness in the breakdown of the bicameral mind. *Am. J. Psychol.* 125 95–112. 10.5406/amerjpsyc.125.1.0095

[B70] Sagan C. (1996). *Demon Haunted World: Science as a Candle in the Dark.* New York, NY: Ballantine Books.

[B71] Seigel J. (2005). *The Idea of the Self: Thought and Experience in Western Europe Since the Seventeenth Century.* Cambridge: Cambridge University Press 10.1017/CBO9780511818141

[B72] Seligman M. E. Steen T. A. Park N. Peterson C. (2005). Positive psychology progress: empirical validation of interventions. *Am. Psychol.* 60 410–421. 10.1037/0003-066X.60.5.410 16045394

[B73] Sheppard G. W. Schulz W. E. (2007). *Code of Ethics.* Ottawa, ON: Canadian Counselling and Psychotherapy Association.

[B74] Skinner B. F. (1974). *About Behaviorism.* New York, NY: Vintage Books.

[B75] Sperry R. W. Gazzaniga M. S. Bogen J. E. (1969). “Interhemispheric relationships: the neocortical commissures; syndromes of hemisphere disconnection,” in *Handbook of Clinical Neurology* Vol. 4 eds Vinken P. J. Bruyn G. W. (Amsterdam: North-Holland Publishing) 273–290.

[B76] Stefflre B. Matheny K. (1968). *The Function of Counselling Theory.* New York, NY: Houghton-Mifflin.

[B77] Strauss C. (2009). “The culture concept and the individualism-collectivism debate: dominant and alternative attributions for class in the United States,” in *Culture, Thought, and Development* eds Nucci L. P. Saxe G. B. Turie E. (Mahwah, NJ: Lawrence Erlbaum Associates) 85–114.

[B78] Taylor S. E. (1989). *Positive Illusions: Creative Self-deception and the Healthy Mind.* New York, NY: Basic Books.

[B79] Trehub A. (2007). Space, self, and the theater of consciousness. *Conscious. Cogn.* 16 310–330. 10.1016/j.concog.2006.06.004 16877006

[B80] Trimpey J. (1996). *Rational Recovery: The New Cure for Substance Addiction.* New York, NY: Pocket Books.

[B81] Warwar S. Greenberg L. S. (2000). “Advances in theories of change and counselling,” in *Handbook of Counselling Psychology* 3rd Edn eds Brown S. D. Lent R. W. (New York, NY: Wiley and Sons) 571–600.

[B82] White L. (1969/1990). “Four stages in the evolution of minding,” in *From Sentience to Symbols: Readings on Consciousness* eds Pickering J. Skinner M. (Toronto, ON: University of Toronto Press) 173–182.

[B83] Wilson E. O. (1999). *Consilience: The Unity of Knowledge.* New York, NY: Vintage Books.

[B84] Wilson G. T. (2014). “Behavior therapy,” in *Current Psychotherapies* 8th Edn eds Corsini R. J. Wedding D. (Toronto, ON: Nelson) 223–262.

[B85] Wolford G. Miller M. B. Gazzaniga M. S. (2000). The left hemisphere’s role in hypothesis formation. *J. Neurosci.* 20 RC64. 10.1523/JNEUROSCI.20-06-j0003.2000 PMC6772516 10704518

[B86] Wu M. (2017). The process of self-cultivation and the mandala model of the self. *Front. Psychol.* 8:24. 10.3389/fpsyg.2017.00024 28174544 PMC5259693

